# Multi-stable oscillations in cortical networks with two classes of inhibition

**DOI:** 10.1371/journal.pcbi.1014391

**Published:** 2026-06-09

**Authors:** Arnab Dey Sarkar, Bard Ermentrout

**Affiliations:** Department of Mathematics, University of Pittsburgh, Pittsburgh, Pennsylvania, United States of America; CRM: Centre de Recerca Matematica, SPAIN

## Abstract

In the classical view of cortical rhythms, interactions between excitatory pyramidal neurons (E) and inhibitory parvalbumin-expressing interneurons (I) are sufficient to generate gamma- and beta-band oscillations. However, it is now well established that multiple inhibitory interneuron subtypes exist and that they play important roles in the generation and modulation of these rhythms. In this paper, we develop a spiking network model consisting of populations of E, I, and an additional interneuron type, somatostatin-expressing neurons (S), which receive excitation from the E cells and inhibit both the E and I populations. The S cells are further modulated by a third inhibitory subtype, vasoactive intestinal peptide (VIP) neurons, which receive inputs from other cortical areas. We reduce the spiking network to a system of nine differential equations that describe the mean membrane potential, firing rate, and synaptic conductance for each population. Using this reduced model, we identify a wide range of parameters that exhibit multiple coexisting rhythms. Employing tools from nonlinear dynamics, we then explore the roles of the two classes of inhibition, as well as VIP modulation, in shaping the properties of these rhythms.

## Author summary

Rhythmic dynamics in the cortex are crucial for information processing, sensory integration, and cognition. In this paper, we examine a model network consisting of a population of excitatory neurons and two distinct populations of inhibitory neurons. We show that the interactions between these three populations give rise to multiple coexisting rhythms. We also present a reduced mean-field model that captures the macroscopic dynamics of the network and reproduces the key multistable behaviors observed in the spiking simulations. Our computational model may provide a mechanism for the experimental appearance of multiple rhythms in the same cortical circuit.

## Introduction

Neuronal oscillations in the cortex span multiple frequency bands and are thought to play distinct roles in information processing and cognitive function [1,2]. Gamma rhythms (approximately 30–80 Hz) are typically associated with local circuit synchronization, sensory processing, and fast excitation–inhibition interactions, often mediated by parvalbumin interneurons [3–5]. Beta rhythms (approximately 13–30 Hz) are more heterogeneous and are commonly subdivided into beta1 (approximately 13–20 Hz) and beta2 (approximately 20–30 Hz) bands; beta1 oscillations have been linked to long-range communication, motor control, and short-term memory maintenance [6,7], while beta2 rhythms are often associated with local cortical circuitry and inhibitory feedback interactions [8,9]. Increasing evidence suggests that interactions between distinct inhibitory interneuron classes, particularly PV and SOM populations, contribute critically to the generation, modulation, and coexistence of these rhythms within the same cortical circuit [10–13].

In recent years, substantial research has focused on the dynamics of **E + I neuron networks**, comprising excitatory pyramidal neurons (E) and inhibitory parvalbumin-positive interneurons (PV, I), that are known to generate oscillatory patterns crucial for various neural functions [14–16]. These studies, particularly those centered on gamma oscillations, have elucidated how the balance of excitation and inhibition shapes neural network activity. However, an increasing body of evidence highlights the importance of other inhibitory interneuron populations, particularly **somatostatin-expressing interneurons (SOM)**, which exhibit distinct temporal properties and connectivity patterns compared to PV [4,5,17].

SOM plays a unique role in cortical circuits by providing dendritic inhibition, thus influencing input integration and network excitability differently from perisomatic inhibition by PV. Their involvement has been implicated in modulating oscillations across various frequency bands, including beta rhythms, which are crucial for motor control, working memory, and attention. Understanding the interaction between excitatory neurons, PV, and SOM is critical to a more comprehensive view of cortical dynamics, especially in contexts where multiple oscillatory regimes coexist and interact [4,18,19].

Using computational modeling and *in vitro* electrophysiological recordings from cortical slices, [8] studies have shown that beta1 and beta2 rhythms arise from overlapping but distinct network dynamics, with excitatory pyramidal neurons primarily supporting beta1, PV enhancing beta2 through strong inhibitory drive, and SOM modulating the phase coupling between the two bands by precisely regulating distal dendritic inhibition and shifting the excitation-inhibition balance to promote cross-frequency coherence [7,9,20–22]. Motivated by these biological observations, we sought to investigate the emergence of multi-rhythmicity in cortical networks using a mathematical model incorporating two distinct types of inhibition: PV and SOM—with vasoactive intestinal peptide (VIP) interneurons acting as a modulatory input that selectively inhibits SOM.

Recent theoretical work has begun to address how multiple inhibitory cell classes interact to control cortical rhythms and network stability. In particular, Edwards et al. [23] constructed a biophysically inspired spiking network model incorporating three distinct neuronal populations: excitatory pyramidal neurons (E), parvalbumin-positive (PV) fast-spiking interneurons, and somatostatin-positive (SOM) interneurons. Their study was motivated by recent *in vivo* findings in the primary visual cortex (V1), suggesting that SOM and PV interneurons have distinct temporal firing patterns and phase-locking behavior during gamma oscillations [4,5]. Through a series of numerical simulations and phase-locking analyses, they revealed that PV and SOM exert qualitatively distinct effects on network dynamics. PV strongly synchronizes excitatory neurons via perisomatic inhibition, facilitating high-frequency gamma rhythms (40–80 Hz), while SOM contributes to slower inhibitory feedback loops that modulate gain and network stability. Crucially, their work demonstrated that alterations in SOM-PV-E connectivity can give rise to rich dynamical regimes, including asynchronous irregular states, bistability, and multirhythmic oscillations. These findings align with earlier computational predictions [9,20,22], and provide new insight into how SOM may gate transitions between different oscillatory modes.

Recent work by Tahvili et al. [10] further advanced this line of research with the CAMINOS model, which dissected the causal contributions of PV and SOM interneurons. Their study demonstrated that PV is critical for controlling oscillation frequency and maintaining stability, while SOM regulates amplitude and promotes slower rhythms. Importantly, they showed that balanced ratios of PV and SOM yield the most stable cortical dynamics, whereas dominance of either class can destabilize the network, sometimes leading to pathological activity. This highlights how interneuron diversity not only enables rhythm generation but also safeguards against instability. Complementary work [11] systematically analyzed all possible E–PV–SOM circuit motifs, identifying a taxonomy of oscillatory states—including theta-nested gamma, stable beta, and theta-locked bursting—that emerge only in networks with multiple interneuron subtypes. Their motif-level classification provides a broad map of circuit behaviors that complements our mechanistic focus on multistability and switching. While the CAMINOS model [10] focuses on the causal roles of PV and SOM interneurons in generating and stabilizing gamma oscillations, our work emphasizes the global bifurcation structure of multi-population inhibitory circuits. In contrast to CAMINOS, which primarily investigates oscillation generation and network stability through spiking simulations, we employ an exact mean-field reduction and systematic bifurcation analysis to characterize multistability, isolated branches of limit cycles, and quasiperiodic dynamics. Thus, whereas CAMINOS elucidates the mechanistic origin of oscillations using mainly stochastic gamma oscillations, our study reveals how multiple inhibitory classes organize the coexistence and switching of distinct rhythmic states.

Inspired by this framework, we extend their approach by implementing a reduced mean-field model that captures the macroscopic dynamics of such three-population networks. Building on [24], we incorporate two classes of inhibitory neurons—PV and SOM—and investigate their role in shaping network rhythms across a range of input conditions and connectivity motifs. In contrast to previous two-population models [25–27], our formulation allows a deeper analysis of the interactions between multiple inhibitory motifs and their impact on spectral composition, phase locking, and cross-frequency coupling.

The primary objective of this study is to explore the dynamics of a three-population neuronal network consisting of **excitatory neurons (E)**, **parvalbumin interneurons (I)**, and **somatostatin interneurons (S)** by building and analyzing **population-level models** and **spiking network models**. We aim to investigate how the interplay between these three populations influences network oscillations and bifurcation structures, which may provide insight into cross-frequency interactions and bistability observed in experimental studies.

The paper is organized as follows. We begin by introducing a spiking model for the three populations (E, I, S) that is based on the quadratic integrate-and-fire/theta neuron formulation. We show that there can be two distinct coexisting rhythms when the excitatory cells are provided with appropriate external drive. The spiking model allows us to explore the phase relationships between the population rhythm and the spikes as well as the synchronization of the neurons and how it differs between the two rhythms. We show that transient stimuli to different populations of neurons enable us to robustly switch between the two rhythms. To better understand the multi-rhythmicity and the qualitative differences between the two distinct rhythms, we employ an exact mean-field reduction that is composed of 9 ordinary differential equations. We use this reduced system to study the importance of the two different inhibitory populations and how they each contribute to the synchrony and properties of the network. We find regimes in parameter space where there are theta-modulated fast rhythms and verify that they occur in the spiking model. We also study how VIP inhibition of the SOM cells affects the network rhythmicity.

## Results

### Spiking model

**Fig 1:**
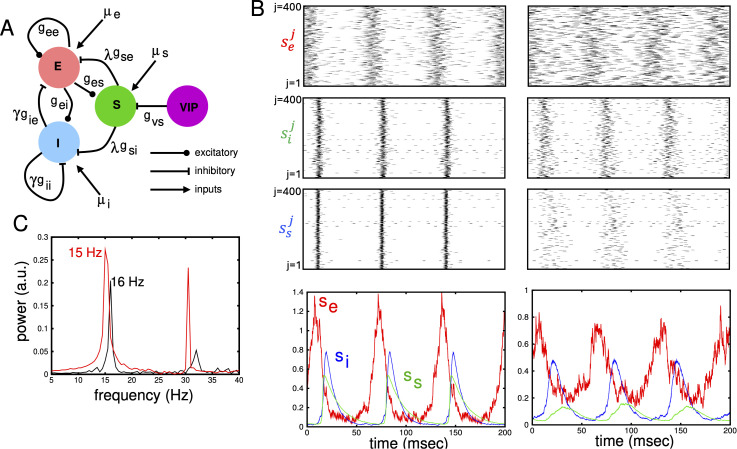
**(A)** Model circuit showing connectivity between excitatory pyramidal cells **(E)**, inhibitory parvalbumin cells **(I)**, inhibitory somatostatin cells **(S)**, and VIP inhibitory cells that modulate the excitability of the S cells. **(B)** Behavior of the network for default parameters with $\mu_{e}=1.25$ showing two distinct rhythms in the same network obtained by starting from different initial states. We label them “big” and “small” limit cycles reflecting the peak amplitude of *s*_*e*_(*t*), the excitatory synaptic activity. Top rows show individual synaptic activity of each neuron in the three populations for the two rhythms and bottom row shows the average of these. **(C)** Power spectra for the two rhythms showing peaks at 15 and 16 Hz.

We have created a network with excitatory cells (E) and three types of inhibition (I, S, VIP) as shown in Fig 1A [12,13,23]. The VIP is represented as constant negative bias modulating the S cells. Each E, I, S population is modeled by 400 all-to-all connected quadratic integrate-and-fire neurons (QIF) with connections mediated by exponentially decaying synapses (see Methods). Each cell in the network receives a constant bias current, common among neurons within the population (e.g., $\mu_{e}$) as well as a constant small random input taken from a Cauchy distribution. The main difference between the S cell population and the I cell population is that the S synapses decay more slowly [23] and their connectivity pattern is different (see Table 2 for a complete list of parameters.) Since the VIP input to the S cells is modeled here as being strictly modulatory, in the simulations and analysis, we incorporate it into $\mu_{s}$, the input to the S cells.

In Fig 1B, we drive the excitatory population with a tonic input and find, depending on how the network is initialized, two distinct rhythms that are close in frequency (15 vs 16 Hz) but differ a great deal in their coherence and amplitude. In the left column (labeled “big limit cycle”), we show rasters of the three populations as well as the population synaptic response (*s*_*e*_,*s*_*i*_,*s*_*s*_) over a 200-millisecond period. The excitatory population is dispersed, but there is a clear rhythm (top and bottom left). Both inhibitory populations show very tight coherence as seen in both the rasters and the population responses. A second rhythm can be found by starting the network with different initial conditions. This rhythm is shown in panel B in the right column and will be referred to as the “small limit cycle”. This slightly higher frequency rhythm is smaller in amplitude and, in particular, the S population fires very weakly with a large spread. Panel C shows the power spectra of *s*_*e*_(*t*) taken from 2 seconds of simulation. The lower-frequency rhythm (15 Hz; left panel in C) exhibits higher spectral power at the fundamental frequency as well as at its higher harmonics. The spectral peaks for the low amplitude 16 Hz rhythm vanish beyond the second harmonic, whereas they persist across multiple higher harmonics for the higher-power (15 Hz) rhythm (not shown). Consequently, the 15 Hz rhythm displays a pronounced second harmonic, while the 16 Hz rhythm does not. We want to emphasize two points: (i) these rhythms do not appear concurrently, rather they are both stable attractors; and (ii) the parameters for both rhythms are the same; this is a multistable system.

**Fig 2:**
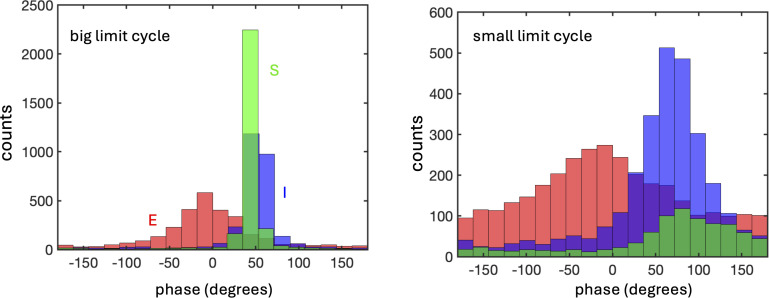
Spike phase relationships to the surrogate local field potential, *s*_*e*_(*t*), for E, I, and S populations for big (left) and small (right) limit cycles with excitation (E) in red, PV (I) in blue, and SOM (S) in green.

In Fig 2, we illustrate the relationships between the spike times of the three populations with *s*_*e*_(*t*) which we take as a surrogate for the local field potential. As has been found in other experimental and theoretical papers [4,10], the two inhibitory classes have different phase relationships. There are differences between the big and small limit cycle oscillations with respect to the number of spikes, how synchronous they are, and the relative phases of spiking. In Table 1, we quantify the differences between the two rhythms. In the first column, we display the Kuramoto order parameter (see Methods) which measures the degree of synchronization; OP = 1 for perfect synchrony and OP = 0 for complete asynchrony. The OP is consistently higher for all three populations in the big limit cycle with a three-fold increase in synchronization for the excitatory cells. The phase and time-shift of the three populations are shown in the next two columns (Angle, Δt). In the big limit cycle, the SOM cells fire on average before the PV cells while the situation is reversed for the small limit cycle. This is also evident in the histograms shown in Fig 2. In the last column, we show the average firing rate of the cells in each population. Except for the SOM cells in the small limit cycle, each neuron fires roughly once per cycle.

**Table 1:** Synchronization (Kuramoto order parameter, OP), mean phase delay relative to the peak of *s*_*e*_, corresponding time delay Δt, and mean firing rate for each population in the big and small limit cycles. All quantities reported in Table 1, including the Kuramoto order parameter and mean firing rate, are computed as time averages over one period of the limit cycle after discarding transients. In particular, the mean firing rate is obtained by averaging *a*_*e*_(*t*) over the cycle, and the Kuramoto order parameter is averaged over time to quantify the overall level of synchrony.

	Big Limit Cycle (15 Hz)				Small Limit Cycle (16 Hz)			
Pop.	OP	Angle (°)	Δt (ms)	Rate (Hz)	OP	Angle (°)	Δt (ms)	Rate (Hz)
E	0.61	-5.26	-0.97	16.12	0.20	-16.17	-2.81	19.27
I	0.85	51.8	9.60	16.67	0.63	69.5	12.06	17.15
S	0.92	44.5	8.24	16.33	0.50	96.9	16.82	5.07

### Mean field models of neural populations

To uncover the mechanisms for this multistability, the conditions underlying it, and to discover other behaviors of the network, we construct an exact mean-field model in the limit as the number of neurons in each population goes to infinity. Montbrió et al. [24] developed a powerful method for reducing populations of quadratic integrate-and-fire (QIF) neurons to low-dimensional models that involve only differential equations for the mean firing rate, the mean potential, and the synaptic kinetics. Their work was based on an equivalence between the QIF and the theta-model [28] and an application of the Ott–Antonsen ansatz [29]. Since these important papers, there have been many subsequent papers extending their approach to other types of neuronal networks [30,31].

In the Materials and Methods, we briefly sketch the derivation of the mean-field model that leads to the following equations:


$$\begin{array}{c} \tau_{m,e}\dot{a_{e}}=2a_{e}b_{e}+\Delta_{e} \end{array}$$ (1a)

$$\begin{array}{c} \tau_{m,e}\dot{b_{e}}=b_{e}^{2}-a_{e}^{2}+\mu_{e}+g_{ee}s_{e}-\gamma g_{ie}s_{i}-\lambda g_{se}s_{s} \end{array}$$ (1b)

$$\begin{array}{c} \tau_{e}\dot{s_{e}}=-s_{e}+a_{e}/\pi \end{array}$$ (1c)

$$\begin{array}{c} \tau_{m,i}\dot{a_{i}}=2a_{i}b_{i}+\Delta_{i} \end{array}$$ (1d)

$$\begin{array}{c} \tau_{m,i}\dot{b_{i}}=b_{i}^{2}-a_{i}^{2}+\mu_{i}+g_{ei}s_{e}-\gamma g_{ii}s_{i}-\lambda g_{si}s_{s} \end{array}$$ (1e)

$$\begin{array}{c} \tau_{i}\dot{s_{i}}=-s_{i}+a_{i}/\pi \end{array}$$ (1f)

$$\begin{array}{c} \tau_{m,s}\dot{a_{s}}=2a_{s}b_{s}+\Delta_{s} \end{array}$$ (1g)

$$\begin{array}{c} \tau_{m,s}\dot{b_{s}}=b_{s}^{2}-a_{s}^{2}+\mu_{s}+g_{es}s_{e} \end{array}$$ (1h)

$$\begin{array}{c} \tau_{s}\dot{s_{s}}=-s_{s}+a_{s}/\pi \end{array}$$ (1i)

For each population, the variable *a* is proportional to the mean firing rate, *b* is the mean voltage, and *s* is the synaptic output of each population. We have included two parameters, γ and λ, which will allow us to vary the influence of the respective I and S inhibitory populations. In keeping with the known circuitry [13], we assume that the S population receives excitation and inhibits both the E and I populations but receives no inhibition from either I or S populations. In addition to differences in connectivity between I and S, we have also made the synaptic decay for S slower, $\tau_{s}=15$ msec vs. $\tau_{i}=7.5$ msec [23]. The VIP population is not explicitly modeled here, but rather will be considered as tonic inhibitory drive to the S population (here, the parameter $\mu_{s}$). In the mean-field model, constant steady states (equilibria) for the system represent *asynchronous* activity in the spiking network, while oscillations (limit cycles) represent synchronous rhythmic behavior for the spiking model (see Fig 1B).

**Fig 3:**
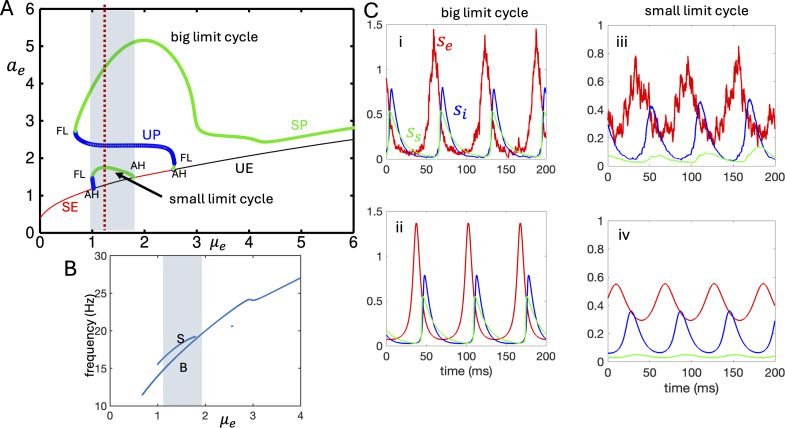
Behavior of the mean-field model compared to the spiking model. **(A)** Bifurcation diagram showing the maximum of *a*_*e*_(*t*) as a function of the excitatory drive, $\mu_{e}$. Solid red (black) lines represent stable (unstable) equilibrium points (SE, UE), and thick green (blue) lines are stable (unstable) periodic solutions (SP, UP). The dashed vertical line indicates $\mu_{e}=1.25$, where there are two stable distinct periodic solutions. Six special points are indicated in the diagram: *Fold of limit cycles* (FL), where a stable (green) and an unstable (blue) oscillation collide and annihilate each other; and the *Andronov–Hopf* bifurcation (AH), where an equilibrium changes stability and either gives rise to a stable oscillation (supercritical AH) or destabilizes through interaction with an unstable oscillation (subcritical AH). The lightly shaded region depicts parameters where there are two distinct oscillations: the “big limit cycle” (larger *a*_*e*_) and the “small limit cycle” (smaller *a*_*i*_). **(B)** Frequency of the two oscillations as a function of $\mu_{e}$. The small (S) limit cycle has a slightly higher frequency than the big (B) limit cycle. **(C)** Comparison of the synaptic variables, $s_{e},s_{i},s_{s}$, for the mean-field and the spiking models. Excitatory is red, inhibitory PV is blue, and inhibitory SOM is green. **(i)** *s*_*j*_(*t*) for the spiking model on the big limit cycle; (ii) mean-field; **(iii)** *s*_*j*_(*t*) on the small limit cycle for the spiking model; (iv) the mean-field.

We have chosen our baseline parameter set (Table 2) such that both the (*E*, *I*) and (*E*, *S*) systems produce limit cycle oscillations when the drive to the excitatory cells, $\mu_{e}$, is sufficiently strong. With all parameters fixed ($\lambda =1,\gamma =0.85,\mu_{s}=-2$), we vary $\mu_{e}$, the drive to the excitatory neurons, and examine what happens in the mean-field model. Fig 3A shows the maximum values of *a*_*e*_(*t*) as $\mu_{e}$ varies between 0 and 6. There are four classes of solutions: (i) red curves are *stable equilibria*; (ii) black curves are *unstable equilibria*; (iii) green curves show the maximum values of *stable oscillations*; and (iv) blue curves show maximum values of *unstable oscillations*. Note: we have chosen to show only the maximum values of the variable *a*_*e*_ because, in later figures where there are *isolas*, depicting maxima and minima makes the figures very confusing. At low excitatory drive, $\mu_{e}$, only stable equilibria exist corresponding to stable asynchronous activity in the spiking network. Qualitative changes in behavior occur at *bifurcations* where equilibria and limit cycles change stability or appear and disappear. The primary bifurcations we see are: *Andronov-Hopf* (AH) bifurcations and *folds of limit cycles* (FL). At an AH bifurcation, an equilibrium point changes stability and a limit cycle is created or destroyed. The AH bifurcation can be either *supercritical*, in which a stable equilibrium loses stability and gives rise to a small-amplitude stable limit cycle, or *subcritical*, in which an unstable limit cycle collides with and destabilizes an equilibrium. A fold of limit cycles occurs when a stable and an unstable limit cycle collide and annihilate each other. In panel A, we depict three AH points and three FL points. The thin vertical line indicates $\mu_{e}=1.25$, the value used in Fig 1. From this diagram, we can immediately see the range of values of $\mu_{e}$ where there exist two distinct stable limit cycle oscillations for the same value of $\mu_{e}$. In the bifurcation diagram, each stable limit cycle is represented by a green curve corresponding to the maximum value of *a*_*e*_ along that cycle. For a fixed value of $\mu_{e}$, drawing a vertical line intersects each stable limit cycle. When the vertical line intersects two distinct pairs of green curves, the system possesses two coexisting stable limit cycles at the same parameter value. We refer to this regime as *bi-rhythmicity*, where the limit cycle with the smaller (larger) maximum value of *a*_*e*_ is termed the small (big) limit cycle, respectively. We use “big” and “small” only in the case where there is coexistence of the rhythms in order to distinguish them. The lightly shaded region in panel A shows the primary parameter set where bi-rhythmicity occurs. It is delineated by the region of stability of the smaller limit cycle, which is completely within the region of the big limit cycle. There is a very small additional region of bi-rhythmicity between the right-most AH and FL. The big limit cycle shows a sharp drop in amplitude (coherence in the spiking model) as $\mu_{e}$ increases from 2 to 3 and a second drop around $\mu_{e}=4$. Fig 3B shows the frequency of the rhythms as a function of $\mu_{e}$ in the same range. The bi-rhythmic region is seen by noting two different frequencies: the smaller-amplitude limit cycle has a slightly higher frequency. The frequency of both rhythms, with few exceptions, increases with $\mu_{e}$ and at high drives begins to enter the gamma range (about 35 Hz). Fig 3C compares the mean-field values of the synaptic variables, *s*_*e*_, *s*_*i*_, *s*_*s*_, with their counterparts in the spiking model; the left is the big limit cycle and the right is the small (compare Fig 1B, bottom). Note that throughout the paper, we will use *a*_*e*_ in the vertical axis for the one-parameter diagrams. The activity in the spiking model is very “noisy”, thus, for the purposes of comparison, we use *s*_*e*,*i*,*s*_ as this is the low-pass filtered version of the activity. Despite the low number of neurons in the spiking model, the agreement is quite good. In the small limit cycle on the right, the SOM population (green) exhibits very weak activity, as measured by the population-averaged synaptic variable, with an amplitude that is small relative to the corresponding PV (blue) and excitatory (E, red) population activities over time, suggesting that the small limit cycle oscillations in panel A are mainly from the (*E*, *I*) network. We will see that this is the case shortly. Because the E cells are firing relatively weakly and $\mu_{s}=-2$, the S population is only weakly engaged. In contrast, on the big limit cycle, the S cells are fully engaged, and since they also inhibit the I cells, this allows for high-amplitude E activity despite the strong S-to-E inhibition.

**Table 2:** Default parameter values for the network model.

$\mu_{e}$	1.25	$\mu_{i}$	-0.5	$\mu_{s}$	-2
*g*_*ee*_	1.5	*g*_*ei*_	2.0	*g*_*es*_	4.25
*g*_*ie*_	1.0	*g*_*ii*_	0.5	*g*_*is*_	0
*g*_*se*_	2.0	*g*_*si*_	0.5	*g*_*ss*_	0
*t*_*me*_	20	*t*_*mi*_	10	*t*_*ms*_	10
$\tau_{e}$	2.0	$\tau_{i}$	7.5	$\tau_{s}$	15
$\Delta_{e}$	0.1	$\Delta_{i}$	0.1	$\Delta_{s}$	0.1
*p*	0.95	λ	0.85	γ	1

**Fig 4:**
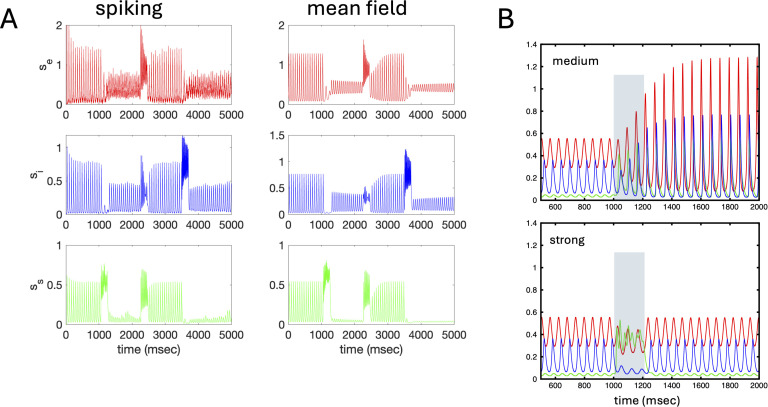
**(A)** Stimuli to different populations of neurons allow for switching between the different oscillations. At *t* = 1000 msec, the SOM cells (green) are given a 200 msec input, which switches the network from the big oscillation to the small one; at *t* = 2050 msec, the E cells (red) are given a stimulus for 200 msec to switch back to the big oscillation; at *t* = 3500 msec, the PV cells (blue) are given a 200 msec stimulus, switching from the big to the small oscillation. **(B)** Top: a 200 msec stimulus of amplitude 1 is given to the SOM population and switches the network from the small to the big oscillation. Bottom: the same stimulus with amplitude 2 is provided and fails to switch the network.

Since our network is bi-rhythmic, we should be able to switch between states by stimulating one or more of the populations. In Fig 4A, we show how 200 msec input pulses can switch between states both in the mean-field and in the spiking network. We initialize both networks so that they are on the big limit cycle. At *t* = 1000 msec, we provide a 200 msec pulse of amplitude 4 to the S cells (green burst). This transiently suppresses both the E and I cells, with suppression of the E cells sufficient to push the system onto the smaller limit cycle. At *t* = 2050 msec, we stimulate the E population with a 200 msec pulse of amplitude 6, which switches it back to the big limit cycle. Finally, at *t* = 3500 msec, we stimulate the I population with a 200 msec pulse of amplitude 3, which suppresses the E cells enough to bring the system to the small limit cycle.

Interestingly, because the S population inhibits both I and E, it is possible to switch the network from the small to the big oscillation by appropriately stimulating the S cells. In Fig 4B (Top), we apply a 200 msec stimulus with amplitude 1 to the S population. This causes the S activity (green) to increase and at the same time suppresses the I activity (blue), but the E activity (red) is affected less. The suppression of I allows the E to escape from the inhibition enough to reach the big oscillation (top panel). However, a stimulus with amplitude 2 (bottom panel), while suppressing the I population even more, also considerably inhibits the E population so that the switch to the big limit cycle is prevented.

So far, we have shown that having two distinct populations of inhibitory interneurons provides a simple mechanism for the appearance of multiple beta frequencies in a coupled network. Some natural questions to ask are: how necessary are two populations; how robust are these behaviors; and are there other types of dynamics. To answer these questions, we have introduced two parameters in the model equations: γ and λ, which scale the inhibition from the I and S cells respectively (see Eq. (1)). For example, increasing γ has the effect of proportionally increasing the I-to-E and I-to-I inhibition. Similarly, setting λ=0 removes the S inhibition from the circuit. In Fig 3A, we saw that the stable states of the system are determined by the AH and FL bifurcations, as these are where oscillations are born and where they die. Thus, in the next few figures, we will co-vary the excitatory drive, $\mu_{e}$, and the scaling factors, λ and γ. Specifically, for each value of, say λ, we will track the values of $\mu_{e}$ where there are AH and FL bifurcations. This will divide the $\left(\mu_{e},\lambda \right)$ plane into regions with different qualitative behaviors. The ensuing diagrams are called *two-parameter bifurcation diagrams*, and we will also replot them in a more intuitive manner where the different types of stable behavior are summarized.

**Fig 5:**
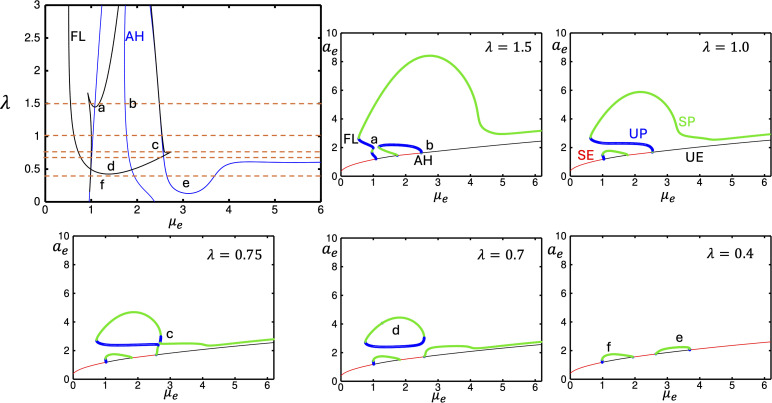
Dependence of dynamics and multi-rhythmicity on SOM (λ). The number of oscillations is organized around the appearance and disappearance through collisions of stable and unstable oscillations (FL, shown as black curves) and the emergence and disappearance of oscillations from equilibria (AH, shown as blue curves). As the degree of SOM inhibition (λ) changes, the dependence of the mean-field system on the excitatory drive ($\mu_{e}$) changes qualitatively. Crossing the black and blue curves changes the number of stable equilibria and oscillations. The letters **a**– **f** in the $\left(\mu_{e},\lambda \right)$ diagram are shown at their corresponding values in the $\left(\mu_{e},a_{e}\right)$ diagrams surrounding the main diagram. SL (UL): stable (unstable) limit cycle; SE (UE): stable (unstable) equilibrium. The label **d** shows an example of an *isola of oscillations* where solutions are not connected to the main branch of equilibria. Once λ falls below about 0.45, there is no bi-rhythmicity. Note: We chose to plot only the maximum values of the limit cycles because, in regions of bistability and especially near isolas, including both maxima and minima made the diagrams difficult to interpret visually. Displaying only the maxima provides a clearer representation of the coexistence and organization of periodic branches without obscuring the overall bifurcation structure.

Fig 5 shows the behavior in the $\left(\mu_{e},\lambda \right)$-plane to see the consequences of varying the degree of S inhibition. To better clarify this diagram, we also show diagrams of *a*_*e*_ vs. $\mu_{e}$ for different values of λ. Starting with λ=0 (just I and E cells), as $\mu_{e}$ increases, the first blue (AH) line (at $\mu_{e}=0.98$) is crossed, giving birth to a small-amplitude limit cycle which persists until the second blue curve is crossed and this limit cycle is lost. (We do not show this plot, but it is very similar to the λ=0.4 case at the label **f**.) As we increase λ, it next crosses a pair of AH (blue) curves producing another small-amplitude stable oscillation. This is shown in the λ=0.4 panel. The point **e** indicates the right-most AH point. A very interesting bifurcation occurs as we further increase λ past around 0.45 near the point **d**. The FL (black) curves are crossed twice indicating the presence of an *isola* of limit cycles. That is, there is an isolated pair of stable and unstable limit cycles that can be seen in the λ=0.7 panel. The small-amplitude oscillation for $\mu_{e}$ between 1 and 2 persists, but the other small-amplitude oscillation (**e** in the λ=0.4 panel) now extends past $\mu_{e}=6$. This can be read off the main diagram since the line λ=0.7 is above the AH curve **e**. Note that there is now bi-rhythmicity between the small limit cycle and the isola. As λ further increases, the isola merges with the right-most small-amplitude oscillation (**c** in the λ=0.75 panel). Further increases in λ lead to dynamics that are qualitatively like those in Fig 3A and the λ=1 panel in the present figure. Increasing λ to 1.5 merges the big limit cycle and the small limit cycle on the left at points **a, b**. Note that we have not indicated whether the AH is sub- or supercritical. However, that information can be inferred from the FL curves that are associated with a sub-critical AH.

**Fig 6:**
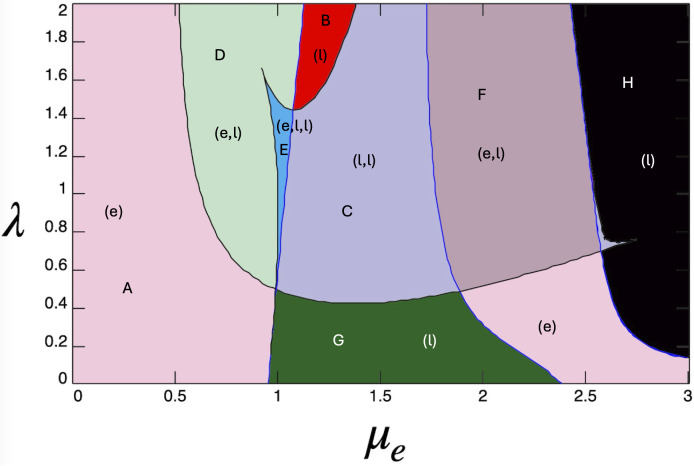
Two-parameter phase-diagram showing how the curves in Fig 5 divide the $\left(\mu_{e},\lambda \right)$-plane into regions with specific numbers of attractors. The letters in parentheses indicate the distinct stable dynamics. For example, in region E, (e,l,l) means there is a stable equilibrium and two stable limit cycles. Regions B, G, and H all contain just one stable limit cycle. Unstable dynamics are not indicated in the figure. As in Fig 5, the regions are separated by black (FL) and blue (AH) curves.

To better clarify the qualitative states of the network, we use the AH and FL curves to divide the $\mu_{e}-\lambda$ plane into distinct regions where there are different attractors. Fig 6 shows this diagram using different colors to aid in separating the regions. Within the labeled regions, we indicate the *stable* states in parentheses. For example, in region E, we have (e,l,l). This means that there are two stable limit cycles (l,l) and one stable equilibrium (e). In regions B, G, H, there is just one stable limit cycle, but we have colored them differently as they are separate “branches” of oscillations. In region B the sole attractor is a big oscillation and corresponds to the limit cycles seen in the λ=(1.47,3) panels in Fig 5. In region G the sole oscillation corresponds to the small-amplitude rhythm shown at the point **f** where λ=0.4 in Fig 5, while region H contains the small-amplitude oscillation seen in all the panels in Fig 5 when $\mu_{e}$ is large. The large region C shows values of $\left(\mu_{e},\lambda \right)$ where there is bi-rhythmicity. In region A, only a single stable equilibrium occurs. We can use the AH, FL curves to understand transitions from one region to another. For example, in the transition from C to F [(l,l) to (e,l)], the blue AH curve is crossed and a small-amplitude oscillation is absorbed by an equilibrium which becomes stable. In the transition from C to B [(l,l) to (l)], a FL curve is crossed indicating that a stable and unstable limit cycle collided and disappeared. Finally, in the transition from C to G, the isola is lost, leading to a single small-amplitude oscillation.

**Fig 7:**
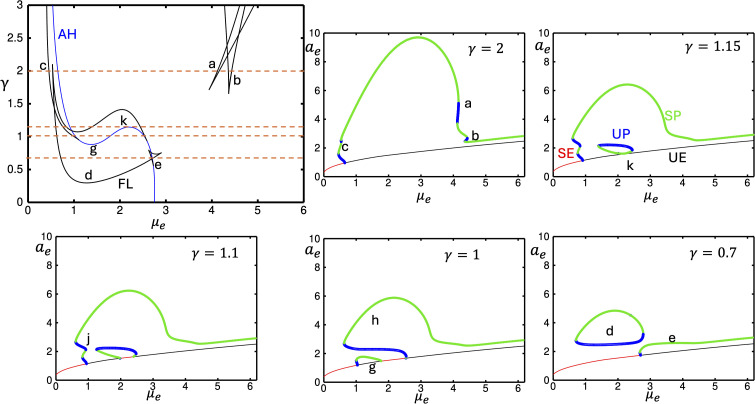
Dependence of dynamics and multi-rhythmicity on PV (γ). See Fig 5 for explanation of curves. If γ falls below about 0.3, multi-rhythmicity is lost. Letters in the $\left(\mu_{e},\gamma \right)$-plane correspond to those in the surrounding $\left(\mu_{e},a_{e}\right)$-plane.

Fig 7 illustrates the behavior in the $\left(\mu_{e},\gamma \right)$-plane, focusing on the consequences of varying the degree of PV (I) inhibition. To further clarify this diagram, we also present graphs of *a*_*e*_ versus $\mu_{e}$ for different values of γ. Starting with γ=0 (where only S and E cells are present), as $\mu_{e}$ increases, the system crosses the first blue supercritical AH bifurcation line at $\mu_{e}=2.7$, leading to a small-amplitude limit cycle. Although this plot is not shown, it closely resembles the branch labeled **e** in the γ=0.7 plot. High values of drive to the E cells are needed to engage the S cells as they have a higher threshold for activation than do the I cells in our model due to the VIP inhibition. Contrast this to the emergence of small-amplitude limit cycle in the EI system in Fig 5 at λ=0 for much lower values of $\mu_{e}$. As γ increases, an isola (**d**) emerges when a horizontal branch crosses two FL curves around γ=0.4 in the two-parameter diagram. As γ increases, a pair of AH bifurcations occur, leading to a small branch of limit cycles (seen in the γ=1 diagram for $\mu_{e}$ roughly between 1 and 2 and labeled **g**). The isola (**d**, γ=0.7) merges with this branch of oscillations ($\mu_{e}>2.75$) to form one big branch (labeled **h** in the γ=1 diagram). Between γ=1 and γ=1.1, the big limit cycle (**h**) collides with the small branch (**g**) to form one branch (**j**). As γ increases slightly beyond γ=1.1, the AH blue curve is crossed (**k** in the $\mu_{e}-\gamma$ diagram), producing another isola (**k** in the γ=1.15 diagram). With continued increases in γ, the isola shrinks and disappears near γ=1.5. Bi-rhythmicity is observed for $\mu_{e}$ in the interval [1,2] when γ ranges approximately from 0.8 to 1.5.

**Fig 8:**
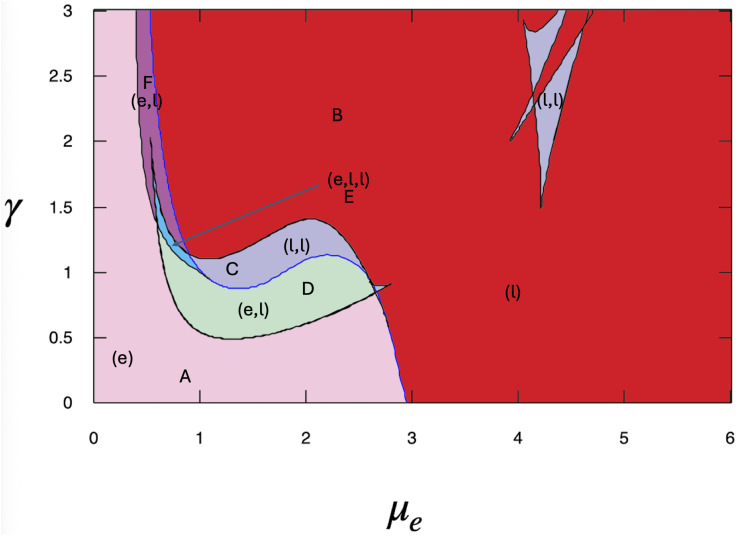
Two-parameter phase-diagram showing how the curves in Fig 6 divide the $\left(\mu_{e},\gamma \right)$-plane into regions with specific numbers of attractors. See Fig 6 for explanation.

In Fig 8 we again partition the $\left(\mu_{e},\gamma \right)$-plane into distinct regions. Region E signifies two stable limit cycles and one stable equilibrium. Regions B, D, and F each contain a single stable limit cycle, but are distinguished by color because they represent different oscillatory branches, not all of which coexist with equilibrium points. In Region B, the stable oscillation corresponds to the big limit cycle which subsequently decreases in size; this is consistently seen across all γ panels in Fig 7. Region D features the big limit cycle coexisting with a stable equilibrium, typically occurring for γ values from 0.5 to 1.3. Region F contains a stable limit cycle whose amplitude increases with $\mu_{e}$ and is visible in the panels where γ ranges from approximately 1.3 to 2 in Fig 7. Regions C and E exhibit bi-rhythmicity; however, Region E also includes a stable equilibrium. Region A is characterized by the presence of a single stable equilibrium. As previously, the AH and FL curves can be used to understand the transitions between these regions. For example, the transition from Region C to Region B (from (l, l) to (l)) occurs when the system crosses a black FL curve, leading to the disappearance of a small-amplitude oscillation, which is absorbed by an unstable limit cycle. Similarly, the transition from Region C to Region D occurs when a small-amplitude stable limit cycle collides with an equilibrium at the AH bifurcation (blue curve).

In our model and others, the primary role of VIP neuronal inhibition is to modulate SOM neurons [13]; thus, we next explore how altering the input to SOM neurons ($\mu_{s}$) affects the presence and multiplicity of rhythms in the EIS network. We combine inhibition from VIP neurons and tonic drive to SOM cells in a single parameter $\mu_{s}$, so that more negative values of $\mu_{s}$ correspond to stronger VIP input.

**Fig 9:**
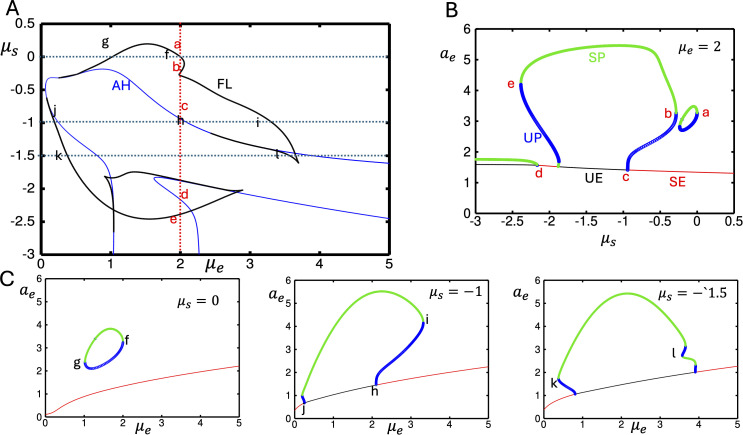
Modulation of SOM by VIP ($\mu_{s}$) has important effects on the dynamics. **A.** As in Figs 5, 6 we show the collisions of stable and unstable limit cycles (FL) in black and the emergence of oscillations from equilibria (AH) in blue. **B.** With $\mu_{e}=2$ fixed (red dashed line in **A)**, we study the behavior as $\mu_{s}$ is increased from −3. Red letters correspond to points in A along the vertical dashed red line. **C.** Dependence on $\mu_{e}$ for different values of $\mu_{s}$ (blue horizontal dashed lines). Black letters correspond to points along the horizontal lines.

**Fig 10:**
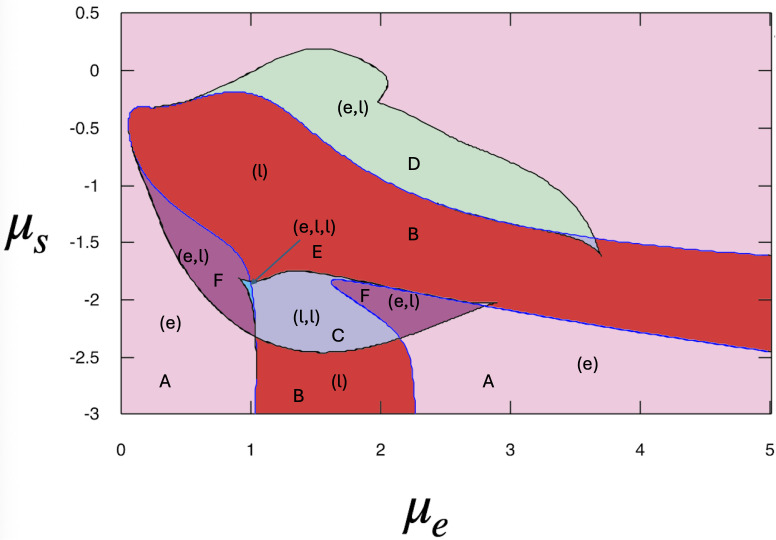
Two-parameter phase-diagram showing how the curves in Fig 9 divide the $\left(\mu_{e},\mu_{s}\right)$-plane into regions with specific numbers of attractors. See Fig 6 for explanation.

In Fig 9 we show the $\left(\mu_{e},\mu_{s}\right)$ two-parameter diagram with sample one-parameter diagrams at different levels of $\mu_{s}$, and in Fig 10 the division of parameters into regions of qualitatively similar behavior. As in the case where we varied the strength of SOM inhibition on E and I populations (Figs 5, 6), varying $\mu_{s}$ has similar effects since it controls the activity of SOM. Fixing $\mu_{e}$, we can follow the effects of VIP inhibition, as shown in Fig 9B where $\mu_{e}=2$. At high VIP inhibition ($\mu_{s}$ very negative), there are only small-amplitude oscillations driven by the E/I subnetwork (below point **d**) as SOM is shut out of the network. Decreasing VIP inhibition produces a stable/unstable pair of large-amplitude limit cycles (region C in Fig 10, point **e** in Fig 9B) and bi-rhythmicity as the SOM population can now interact with the E population to generate rhythms (between the points **d** and **e** in Fig 9B). Further increases in $\mu_{s}$ (decreasing VIP inhibition) increase the activity of the SOM neurons and the small-amplitude oscillation disappears (point **d**). At $\mu_{s}=-1$ (point **b**), the asynchronous state is stabilized leading to bistability between the large-amplitude rhythm and the asynchronous state (between points **c** and **b**). Increases in $\mu_{s}$ lead to the loss of the large-amplitude oscillation (**b**) and, in a small region, an isola emerges **a**. Once $\mu_{s}$ is large, SOM inhibition stabilizes the asynchronous state. Holding $\mu_{s}$ at different levels and varying $\mu_{e}$ (Fig 9B, horizontal lines and Fig 9C) shows examples of isolas of limit cycles and bistability between an oscillatory state and the asynchronous rest state. Bi-rhythmicity (not seen in these three one-parameter diagrams) requires stronger inhibition of SOM (such as $\mu_{s}=-2$ seen in Fig 3A). The black letters in the $\left(\mu_{e},\mu_{s}\right)$-plane, as well as the accompanying one-parameter diagrams, illustrate some examples of multistability between equilibria and oscillations.

**Fig 11:**
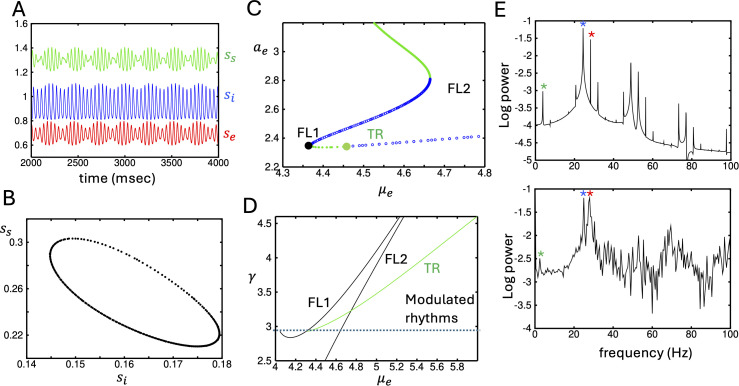
Quasi-periodic dynamics. **(A)** Synaptic dynamics (vertically shifted for clarity) for $\gamma =3,\mu_{e}=4.8$ showing amplitude modulation; (B) values of (*s*_*i*_, *s*_*s*_) whenever *s*_*e*_(*t*) crosses 0.7 from below; (C) zoomed-in one-parameter diagram showing the appearance of the *torus bifurcation* (onset of modulated rhythms, TR); FL1 shows the fold of limit cycles producing oscillations that eventually become the quasi-periodic solutions shown in (A,B) and marked by TR; **(D)** $\left(\mu_{e},\gamma \right)$-diagram showing (green line) where modulated solutions arise. They terminate at the left when the green line runs into the FL1 (black curve). To the right of TR and the left of FL2, there is multistability between modulated rhythms and regular oscillations.; **(E)** Power spectrum of *s*_*e*_(*t*) for the mean-field model (top) and the spiking model (bottom), showing peaks at about 24 and 28 Hz and a small peak at about 3.75 Hz, the modulation frequency.

We have shown that over a wide range of synaptic strengths for SOM and PV cells, our network is capable of producing two *distinct* rhythms that are close in frequency but differ in the degree to which the SOM cells participate and in their degree of coherence (c.f. Fig 3C and Table 1). One common feature in local field potential (LFP) recordings is the occurrence of multiple peaks in the power spectrum. One simple mechanism for multiple peaks is that there are two completely independent oscillatory populations and the LFP represents their summed activity. A more interesting mechanism is that the multiple frequency peaks are all part of the same circuit and represent *quasi-periodic* behavior. Mathematically, quasi-periodic behavior can arise when a limit cycle loses stability to a *torus bifurcation*. We have found that setting γ>3 (very strong PV inhibition) can result in this kind of behavior for our network as $\mu_{e}$ increases. In Fig 11A, we show the synaptic activity of the E, I, and S populations for $\mu_{e}=4.8,\gamma =3$. There is a high-frequency rhythm whose amplitude is slowly modulated with a frequency of 3.75 Hz, indicative of quasi-periodic behavior. To further check whether the dynamics is quasi-periodic, in panel B we plot a dot in the (*s*_*i*_, *s*_*s*_)-plane each time *s*_*e*_ crosses the value 0.7 from below. The result of this plot is an ellipse indicating that the dynamics is filling up a two-dimensional torus. In panel C, we show the bifurcation diagram with γ=3 as $\mu_{e}$ increases. We only show a small window of the diagram in order to focus the reader’s attention on the region near $\mu_{e}=4$. A stable small periodic branch emerges from a fold of limit cycles at $\mu_{e}\approx 4.35$ (**FL1**, in Fig 11C), and this branch loses stability at a torus bifurcation (TR) at $\mu_{e}\approx 4.45$. For $\mu_{e}>4.45$, there is bistability between quasi-periodic behavior and a large-amplitude limit cycle. The latter is lost at FL2. In Fig 11D, we show a two-parameter diagram in the $\left(\mu_{e},\gamma \right)$-plane. Modulated rhythms appear to the right of the green TR curve and for γ above the point where FL1 and TR intersect (γ≈2.9). Multistability between the quasi-periodic behavior and a large-amplitude limit cycle occurs in the small region to the left of FL2 and the right of TR.

In Fig 11E, we show the power spectra for *s*_*e*_(*t*) for $\mu_{e}=4.8,\gamma =3$ for both the mean-field model (top) and the full spiking model (bottom). There are two major peaks at 24.5 and 28.25 Hz and a smaller peak at 3.75 Hz that occurs at the difference between these two frequencies and is responsible for the slow modulation (or beating).

## Discussion

In this paper, we have shown that adding multiple types of inhibitory neurons greatly extends the types of oscillatory dynamics that are possible in cortex-like circuits. Starting with a spiking network composed of coupled quadratic integrate-and-fire neurons, we found that for certain ranges of parameters, there was multi-rhythmicity. That is, there were two distinct rhythmic states, and we can switch between them with appropriate stimuli to the excitatory or inhibitory classes. The range of parameters over which there is bi-rhythmicity is fairly broad, suggesting that such dynamics may be observable in *in vitro* preparations. Since it is possible to selectively stimulate different populations of neurons with optogenetics, it might even be possible to experimentally switch between rhythms. The two distinct beta rhythms, while similar in frequency, differ in their local coherence and in the degree of SOM participation (Fig 3C). This could be visible from the sharpness of the beta band peak in the local field potential. With the availability of SOM-Cre mice, it is now possible to selectively stimulate or inactivate SOM optogenetically and test the dose-dependent activation of SOM seen in Fig 4B. Finally, pharmacological suppression of SOM activity in cortical slices should eliminate one of the peaks in beta.

To facilitate the analysis of this oscillatory circuit, we employed an exact mean-field reduction that produced a nine-dimensional set of ordinary differential equations amenable to a thorough bifurcation analysis. We examined the contribution of the two different classes of inhibition by fixing the conductance parameters and then varying the excitatory drive while at the same time scaling the strength of each inhibitory population onto the other populations (I to E and I to I or S to E and S to I). Figs 6 and 8 show that multi-rhythmicity arises from the interaction of both inhibitory pathways and cannot be sustained by either population alone. The region of multi-rhythmic behavior occurs over a larger range of SOM (S) interactions than for PV (I), as can be seen in the much larger purple area in Fig 6 than in Fig 8. We also found small areas where there is tristability with two stable rhythms and a stable equilibrium. Oscillations appear and disappear via two different mechanisms: (1) Andronov-Hopf bifurcation, where a new oscillation arises from or is absorbed into an equilibrium; (2) folds of limit cycles, when an unstable and stable oscillation pair emerges or disappears. We also found regions where there are isolated branches of limit cycles (isolas), such as in Fig 5 (λ=0.7) or Fig 9 ($\mu_{s}=0.0$). The interaction between the three populations of neurons could provide an alternative explanation for the multiple beta rhythms studied in [9].

VIP inputs into SOM cells have an important modulatory effect on the circuit [10,12,23]; therefore, we also looked at the effects of VIP inputs onto the SOM cells. Here, we treated the input as a constant negative drive, and as would be expected, at high VIP input ($\mu_{s}$ very negative), we essentially remove S from the circuit and bi-rhythmicity disappears. If the VIP input is too weak (that is, $\mu_{s}$ increases), then bi-rhythmicity also disappears.

With strongly enhanced PV coupling strength (roughly 3-fold the default), we found a regime where there is *quasi-periodicity*, Fig 11, manifested as a low-frequency (3 Hz) amplitude modulation of higher-frequency oscillations (25–27 Hz) and two close peaks in the power spectrum. This type of behavior has not been seen in simple E–I networks to our knowledge and could provide a mechanism for theta-gamma coupling [32]. These modulated rhythms occur with high drive to the E cells and strong PV inhibition. The regime where quasi-periodicity occurs (Fig 11D) suggests that pathologically elevated PV activity – as has been proposed for schizophrenia [18,19]– could shift the network from bi-rhythmic to quasi-periodic dynamics with consequences for information processing that remain to be explored.

The present study is similar to a recently published paper [10], in which the authors used a network of exponential integrate-and-fire neurons with E, S, and I cells. One major difference is that their I cells have the same synaptic decay time, while in our model the SOM (S) cells decay slower than the PV (I) cells [23]. Like [10], the frequency of our rhythms was in the lower gamma oscillation range and closer to beta (20–30 Hz); however, with enough drive, we are able to get higher-frequency oscillations such as seen in E/I networks [3]. The “noise” in [10] arises from Poisson inputs into the cell types. In our study, the source of noise comes from the heterogeneity in the external drives that were used so that we could create a mean-field model. Recent work [33] suggests some generalizations of the mean-field equations we studied here are possible when there is Gaussian noise. Like [10], the spike-phase relationships between the SOM and PV cells with the LFP are tighter than those observed experimentally (cf. Fig 1 in [10]). Interestingly, in their study, the PV peak is always earlier than the SOM peak. In Fig 2 and Table 1, SOM leads PV for the big limit cycle but trails PV for the small limit cycle. Because they use a high-dimensional spiking model that has no simple mean-field description, they explored a limited set of parameter values. However, they also interchanged the SOM and PV connectivity to better tease out the contributions of the two populations. [12] used a firing-rate formulation and was aimed at the analysis of gain modulation rather than rhythmicity. [23] found regions of oscillatory dynamics at 20–30 Hz when they drove the SOM populations in their spiking model, which is based on the exponential integrate-and-fire model. Their model included VIP neurons and, because they were driven by the E cells, they are an integral part of the circuit. In our simplified circuit, VIP is just an inhibitory drive to the SOM population. Unlike our model and [10,23] employed a spatially distributed model. A major difference between our results and theirs is that they did not observe oscillations when the SOM population is strongly inhibited. With reduced SOM, we still have regions where there are rhythms such as Fig 5 (λ=0.4).

We should emphasize what is gained by having two populations of inhibition. In classic E/I networks bistability between asynchronous (equilibrium) and rhythmic activity is common. The addition of a second class of inhibition *with distinct synaptic timescales and connectivity* creates a new dynamical degree of freedom leading to new types of behavior including birythmicity and quasi-periodic behavior. Removing SOM from the circuit eliminates the bi-rhythmic regime in our model, suggesting that the longer timescale inhibition is essential for these dynamics and cannot be compensated by increasing PV inhibition.

From a dynamical systems perspective, the coexistence of two inhibitory pathways with distinct time scales introduces competing feedback loops, which enables the emergence of multiple stable oscillatory attractors within the same circuit.

A natural question that arises is whether there are any computational advantages to having a system with multiple stable rhythms. Since oscillatory neural activity has been hypothesized to organize and synchronize local assemblies of neurons [2,6,34], having multiple stable oscillations might allow for the handling of multiple input streams in parallel.

In the present study, our E cells do not have adaptation. In a recent paper [35] we showed that inhibition controlled firing sparsity in E/I networks and that adaptation only played a role when the inhibition was greatly reduced. In the present study, we have two types of inhibition so that even if one type is reduced, the other will compensate. Adaptation was incorporated in [10] with a time constant of 500 msec. Since the time scales of our oscillations are nearly ten-fold faster, the addition of adaptation would effectively be a negative constant current. Our neurons are point neurons and SOM inhibition typically targets dendrites rather than soma. We have given our SOM cells a longer synaptic decay constant to approximate the electrotonic effects of dendritic inputs. One could in principle add a second compartment to the E cells, but it is unclear whether a mean-field model could be derived.

In summary, this paper presented a systematic exploration of the interaction between excitatory and two different inhibitory populations representing parvalbumin and somatostatin subtypes. Through a combination of mathematical modeling, bifurcation analysis, and spiking network simulations, we have provided a deeper understanding of how the balance of excitation, inhibition, and inhibitory modulation governs the complex oscillatory behavior of cortical circuits. Our results may have implications for understanding neural mechanisms underlying cognitive processes and disorders associated with altered beta and gamma oscillations.

## Materials and methods

### Simulations

The spiking model consists of three populations of 400 quadratic integrate and fire neurons:


$$\begin{array}{c} v_{ej}^{\prime }=\frac{1}{\tau_{me}}\left[i_{e}(t)+(v_{ej}{)}^{2}+\mu_{e}+\Delta_{e}\zeta_{je}+g_{ee}s_{e}-\gamma g_{ie}s_{i}-\lambda g_{se}s_{s}\right] \end{array}$$ (2)

$$\begin{array}{c} v_{ij}^{\prime }=\frac{1}{\tau_{mi}}\left[i_{i}(t)+(v_{ij}{)}^{2}+\mu_{i}+\Delta_{i}\zeta_{ji}+g_{ei}s_{e}-\gamma g_{ii}s_{i}-\lambda g_{si}s_{s}\right] \end{array}$$ (3)

$$\begin{array}{c} v_{sj}^{\prime }=\frac{1}{\tau_{ms}}\left[i_{s}(t)+(v_{sj}{)}^{2}+\mu_{s}+\Delta_{s}\zeta_{js}+g_{es}s_{e}-\gamma g_{is}s_{i}-\lambda g_{ss}s_{s}\right] \end{array}$$ (4)

$$\begin{array}{c} s_{e}^{\prime }=-\frac{s_{e}}{\tau_{e}}+\frac{1}{400}\sum_{j=1}^{400}\left(\frac{\tau_{me}}{\tau_{e}}\delta (t-t_{ej})\right) \end{array}$$ (5)

$$\begin{array}{c} s_{i}^{\prime }=-\frac{s_{i}}{\tau_{i}}+\frac{1}{400}\sum_{j=1}^{400}\left(\frac{\tau_{mi}}{\tau_{i}}\delta (t-t_{ij})\right) \end{array}$$ (6)

$$\begin{array}{c} s_{s}^{\prime }=-\frac{s_{s}}{\tau_{s}}+\frac{1}{400}\sum_{j=1}^{400}\left(\frac{\tau_{ms}}{\tau_{s}}\delta (t-t_{sj})\right). \end{array}$$ (7)

where *v*_*zj*_ is the membrane potential of the *j*^th^ neuron (j=1,…,400) in population z∈{e,i,s}, $\mu_{z}$ is the tonic drive to population *z*, *g*_*zw*_ is the coupling strength from population *z* to population *w*, $\tau_{mz}$ is the membrane time constant, $\Delta_{z}$ is the degree of heterogeneity, *s*_*z*_ is the synaptic time course for each population, $\tau_{z}$ is the synaptic decay time constant, *i*_*z*_(*t*) is time-dependent input, and $\zeta_{jz}$ are random numbers drawn from the Cauchy distribution with density function $f(\zeta )=1/[\pi (1+\zeta^{2})]$. The times *t*_*zj*_ are defined as


$$\begin{array}{c} \lim_{t\to t_{zj}}v_{zj}(t)=+\infty . \end{array}$$

When $v_{zj}(t^{-})=+\infty$, *s*_*z*_(*t*) is incremented by $\tau_{mz}/(400\tau_{z})$ and $v_{zj}(t^{+})=-\infty$. For the purposes of simulations, we make the transformation v(t)=tan(θ(t)/2) [28] so that θ=π corresponds to v(t)=+∞ and θ=−π corresponds to the reset to −∞. Note that on the circle, −π and π are identified so that with this transformation all the behavior is continuous. The equation


$$\begin{array}{c} v^{\prime }=a+bv^{2} \end{array}$$

becomes


$$\begin{array}{c} \theta^{\prime }=b(1-\cos\theta )+a(1+\cos\theta ). \end{array}$$

The spiking model is integrated using the Euler method with a time step of 0.02 msec. To test numerical accuracy, we halved our time step and the result did not change. The mean-field model (see main text) is integrated using the CVODE method. All simulations and bifurcation diagrams are created using XPPAUT [36]. Codes will be available on github after publication.

### Spike time statistics

Phase relationships between the spikes and *s*_*e*_(*t*) are found by taking a 1000 msec stretch of data in the spiking model and identifying the peaks of *s*_*e*_(*t*). The phase of a spike occurring at *t*_spike_ in the (E, I, S) populations is determined by finding the closest *s*_*e*_(*t*) peak ($t_{\text{peak}}^{j}$) that precedes *t*he spike and then letting:


$$\begin{array}{c} \theta =360\;\frac{t_{\text{spike}}-t_{\text{peak}}^{\;j}}{t_{\text{peak}}^{\;j+1}-t_{\text{peak}}^{\;j}}. \end{array}$$

To compute the statistics in Table 1, we convert the spike phases $\theta_{j}$ to radians, $\phi_{j}=2\pi \theta_{j}/360$, and compute


$$\begin{array}{c} Z=\frac{1}{M}\sum_{j=1}^{M}\exp(i\phi_{j}), \end{array}$$

where *M* is the total number of spikes counted from the given population in the given oscillation. The Kuramoto order parameter is OP=|Z|, and the average phase shift is $\bar{\phi }=\text{arg}(Z)$. These are converted to degrees and time lag for Table 1.

### Brief derivation of the mean-field model

Here, we sketch the derivation of the mean-field model for completeness (see [24] for complete details). To simplify the derivation, we consider a single population of globally coupled quadratic integrate-and-fire neurons:


$$\begin{array}{c} v_{j}^{\prime }=(v_{j}^{2}+\mu +\Delta \zeta_{j}+gs)/\tau_{m}\equiv J(v_{j},\zeta_{j},s), \end{array}$$

$$\begin{array}{c} s^{\prime }=-s/\tau_{s}+\frac{1}{N}\sum_{j=1}^{N}(\tau_{m}/\tau_{s})\delta (t-t_{j}). \end{array}$$

We take the limit as N→∞ and write the density of the voltages as P(V,ζ,t). The ζ are taken from the Cauchy distribution, $p(\zeta )=1/(\pi (1+\zeta^{2}))$. The density *P* satisfies


$$\begin{array}{c} \frac{\partial P}{\partial t}+\frac{\partial }{\partial V}[J(V,\zeta ,s)\;P]=0. \end{array}$$

The population firing rate is


$$\begin{array}{c} f(t)=\int_{-\infty }^{\infty }p(\zeta )\;\lim_{V\to \infty }J(V,\zeta ,s)P(V,\zeta ,t)\;d\zeta , \end{array}$$

and so


$$\begin{array}{c} s^{\prime }=-s/\tau_{s}+(\tau_{m}/\tau_{s})f(t). \end{array}$$

The key observation that [24] makes is that an explicit form for *P* can be found:


$$\begin{array}{c} P(V,\zeta ,t)=\frac{\alpha (\zeta ,t)}{\pi [(V-\beta (\zeta ,t){)}^{2}+\alpha (\zeta ,t{)}^{2}]} \end{array}$$

provided the complex function w=α+iβ satisfies


$$\begin{array}{c} w_{t}=i[\Delta \zeta +gs-w^{2}+\mu ]/\tau_{m}. \end{array}$$

With the expression for *P*,


$$\begin{array}{c} f(t)=\int_{-\infty }^{\infty }p(\zeta )\;\alpha (\zeta ,t)/(\pi \tau_{m})\;d\zeta . \end{array}$$

The density p(ζ) has poles at ±*i*, so the integral can be evaluated using contour integration, yielding $f(t)=a(t)/(\pi \tau_{m})$ where a(t)=α(−i,t). From this, one finds


$$\begin{array}{c} a^{\prime }=(2ab+\Delta )/\tau_{m}, \end{array}$$

$$\begin{array}{c} b^{\prime }=(b^{2}-a^{2}+\mu +gs)/\tau_{m}, \end{array}$$

$$\begin{array}{c} s^{\prime }=(-s+a/\pi )/\tau_{s}, \end{array}$$

where b(t)=β(−i,t). We remark that *a*(*t*) is proportional to the firing ra*t*e and *b*(*t*) is the mean population voltage.
